# A Rare Case of Congenital Internal Carotid Artery Agenesis and Contralateral Internal Carotid Artery Aneurysm

**DOI:** 10.7759/cureus.23619

**Published:** 2022-03-29

**Authors:** Sanjay Lamsal, Brooke Burkins, Mika Matteo, Jerry Matteo, Taylor S Harmon

**Affiliations:** 1 Radiology, University of Florida College of Medicine, Jacksonville, USA; 2 Radiology, Edward Via College of Osteopathic Medicine, Auburn, USA; 3 Radiology, University of Florida, Gainesville, USA

**Keywords:** cavernous carotid artery aneurysm, magnetic resonance imaging, computed tomography angiography, interventional radiology, pipeline embolization, cerebral digital substraction angiography, transarterial coil embolization, internal carotid artery aneurysm, internal carotid artery, internal carotid artery agenesis

## Abstract

Agenesis of the internal carotid artery (ICA) is a rare congenital entity. This anomaly is typically occult in nature. However, the effects of an incidental discovery secondary to a contralateral ICA aneurysmal rupture can be devastating. The association between agenesis of the ICA and contralateral intracranial aneurysm formation is significantly higher than de novo incidental intracranial aneurysms in the general population. It is important to evaluate the presence of a contralateral intracranial aneurysm in the setting of known agenesis of the ICA. This allows for the performance of prophylactic embolization and characterizes collateral cerebral circulation.

## Introduction

The cervical great vessels, aortic arch, and pulmonary trunk develop from six embryological pharyngeal arches [1-2]. internal carotid artery (ICA) agenesis is a rare discrepancy in the embryological development of the aortic arch and great vessels [3]. There are six main types of congenital ICA agenesis that are documented, the most common involving the left ICA [4-5]. When ICA agenesis occurs, the Circle of Willis allows for collateralization and perfusion of the cerebral vasculature, most often with the contribution of the posterior circulation and persistent fetal origins of the posterior cerebral arteries [6]. There are seven segments of the ICA, which are used to localize any existing ICA pathology [7]. Development of an ICA aneurysm contralateral to a congenitally absent ICA is rare. However, there is a high mortality rate when a contralateral aneurysm is present [8]. It is thought that the development of these aneurysms is acquired and attributed to the pathophysiology explained by the high flow theory [9]. The high flow theory suggests that a higher volume of flow persists through a singular ICA, resulting in abnormal endothelial wall shear stress [10]. The damage to the vessel endothelium initiates remodeling and the production of vasoactive substances, ultimately leading to vessel wall dilation and eventual rupture [10]. Various endovascular and percutaneous interventions may be performed to exclude these ICA aneurysms. 

## Case presentation

**Figure 1:**
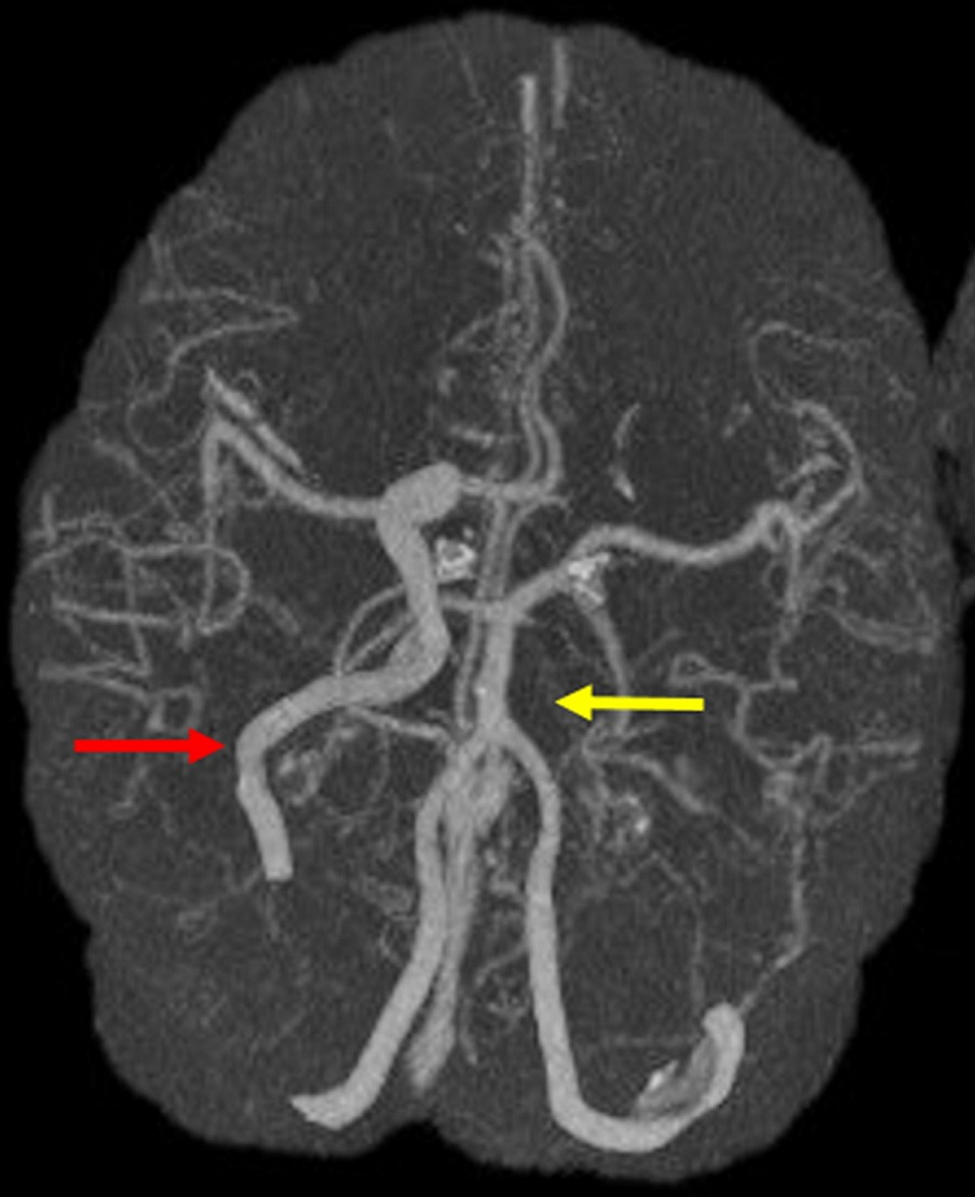
Computed Tomography Angiogram Showing Agenesis of the Left Internal Carotid Artery An axial maximum intensity projection computed tomography angiogram demonstrates agenesis of the left internal carotid artery (yellow arrow). The native right internal carotid artery is shown (red arrow).

**Figure 2:**
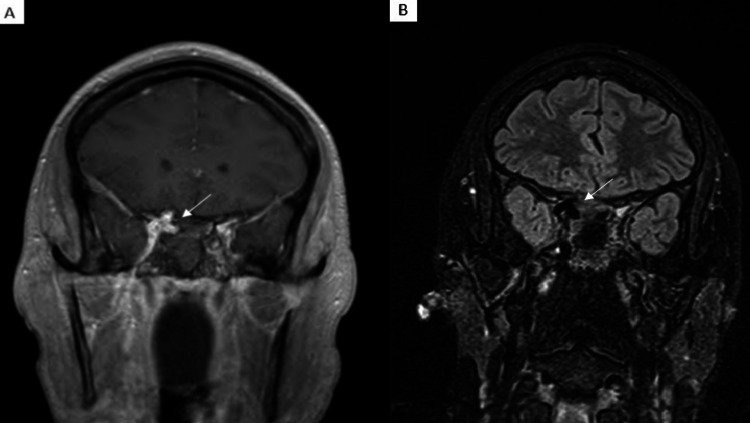
Right Cavernous Internal Carotid Artery Aneurysm Shown on Magnetic Resonance Imaging Coronal T1 post-contrast (A) and coronal T2 fluid-attenuated inversion recovery (FLAIR) sequence (B) magnetic resonance imaging demonstrates a right cavernous internal carotid artery aneurysm (white arrows). The left internal carotid artery flow-void is absent due to agenesis.

The following is a case of a 29-year-old female with a type A agenesis of the left ICA, who presented to the emergency department with intractable pulsatile epistaxis and hypovolemic shock. The patient was initiated on a massive transfusion protocol followed by intubation for airway protection due to rapid deterioration. The initial computed tomography angiogram (Figure 1) and magnetic resonance imaging (Figure 2) of the patient's head revealed a right cavernous ICA aneurysm causing dehiscence and encroachment into the right ethmoid and sphenoid sinuses. The patient was also found to have a congenitally absent left ICA (Figure 1). The presence of this anatomic variant made any surgical option, such as the sacrifice of the carotid artery, a high risk for the development of a large vessel territorial infarct.

**Figure 3:**
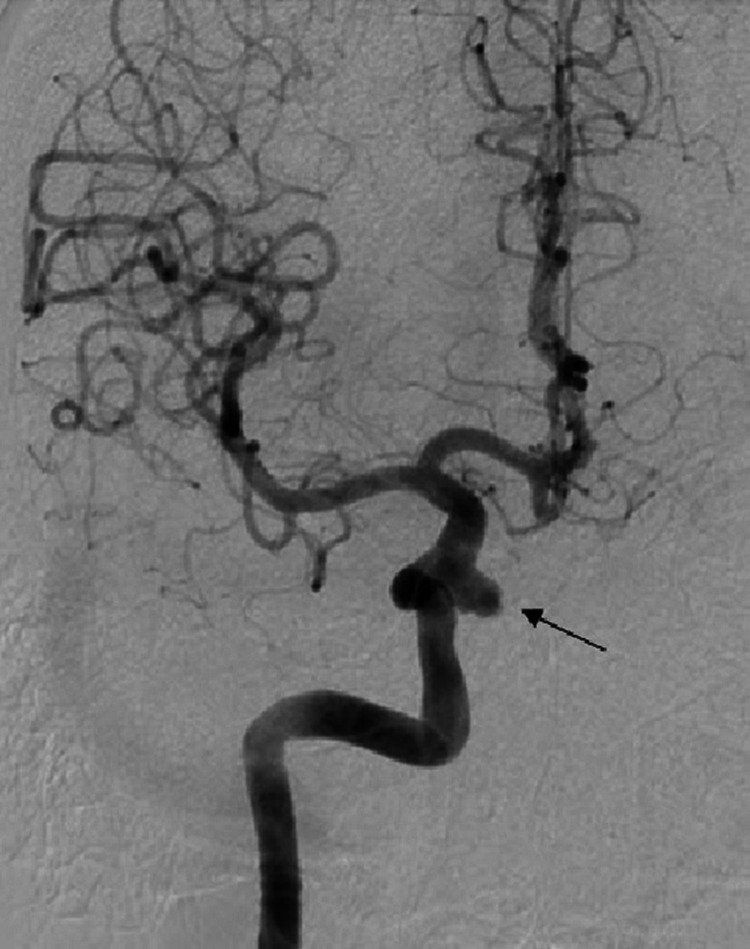
Diagnostic Cerebral Angiogram of a Right Internal Carotid Artery Aneurysm A diagnostic cerebral angiogram through the right internal carotid artery demonstrates a wide-neck aneurysm of the cavernous segment (black arrow).

The interventional radiology service was consulted for angiogram and intervention. An initial angiogram demonstrated the right internal carotid cavernous aneurysm (Figure 3).

**Figure 4:**
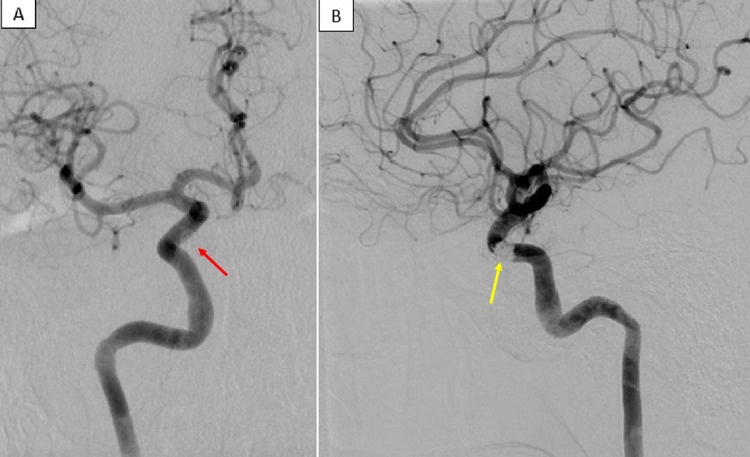
Post-Treatment Cerebral Angiogram After Coil Embolization of a Right Internal Carotid Artery Aneurysm Post-treatment cerebral angiograms of the right internal carotid artery are shown in two planes. The anterior-posterior projection (A) shows proximal and distal opacification of the right internal carotid artery after coil embolization of a cavernous segment aneurysm. The deployed coils are faintly visualized, which exclude the cavernous segment aneurysm (red arrow). The lateral projection (B) shows the deployed coils within the excluded cavernous segment aneurysm, superimposed to a patent right internal carotid artery (yellow arrow). The patent right internal carotid artery perfuses the left anterior cerebral artery through the anterior communicating artery. The patent left posterior fetal circulation perfuses the left middle cerebral artery. These findings confirm type A agenesis of the left internal carotid artery.

Local control of the ruptured right ICA aneurysm was achieved by employing a combination of coils and flow diversion with a pipeline embolization device. A post-treatment angiogram demonstrated complete occlusion of the aneurysmal sac with patent proximal and distal flow within the ICA (Figure 4A). The angiogram also demonstrated filling of the contralateral anterior cerebral artery and middle cerebral artery via the ipsilateral posterior communicating artery (Figure 4B).

Following coil embolization, the patient's epistaxis resolved, and was fluid resuscitated. The patient remained hemodynamically stable in the post-interventional period.

**Figure 5:**
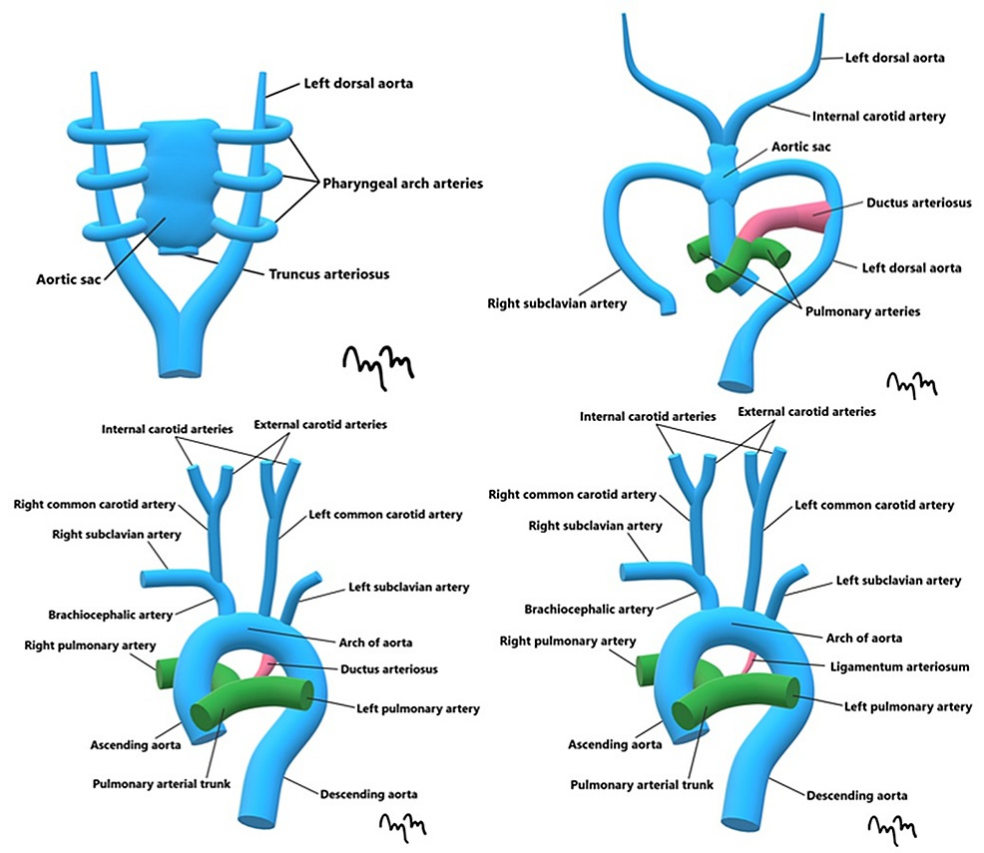
Formation of the Major Mediastinal and Cervical Great Vessels An artist's rendition shows the migration of mesodermal cells within the pharyngeal arches into the aortic sac, eventually forming the mature vasculature.

During the fourth week of embryological development, the six pharyngeal arches begin to develop and are supplied by the pharyngeal arch arteries, also known as the aortic arches (Figure 5).

Mesodermal cells from the pharyngeal arches begin migrating to the aortic sac under the regulation of transcription factor T-Box Transcription Factor 1, connecting the pharyngeal arch arteries to the dorsal aorta of the ipsilateral side [1]. The presence of all six arches is variable, and are not all present at one time. The formation and involution of the first two arches occur before the formation of the fifth and sixth arches [2]. The variability in the regression or persistence of each arch leads to the discrepancy between normal and abnormal anatomy.

**Figure 6:**
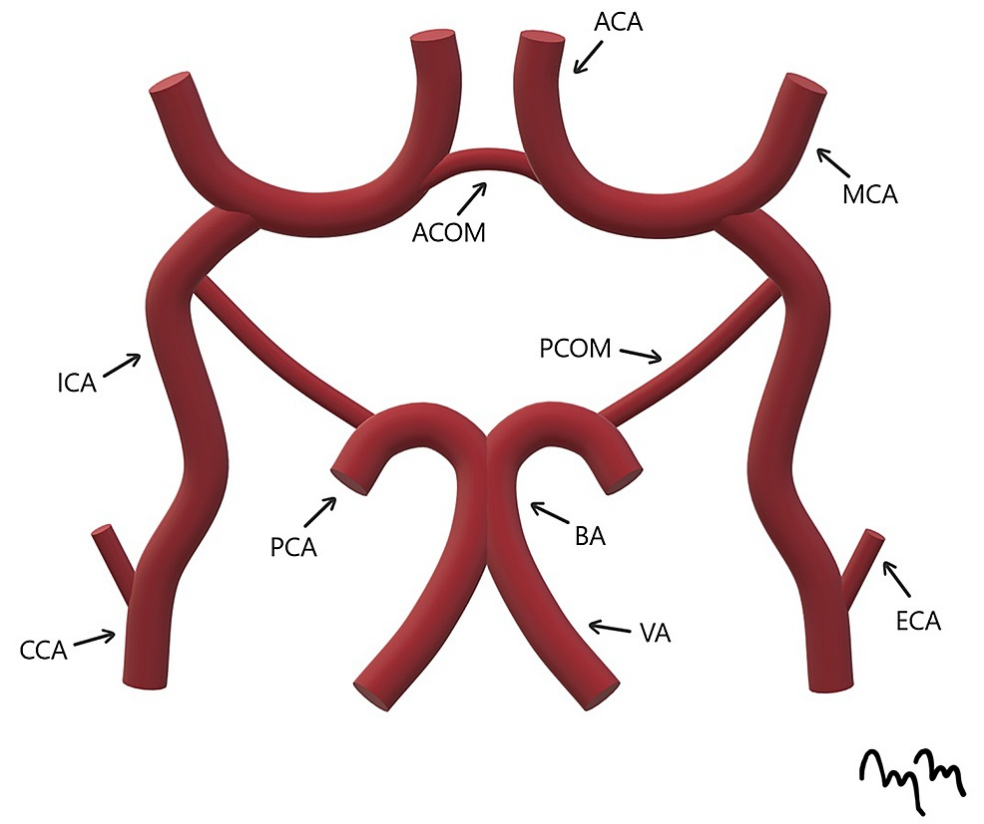
Normal Anatomy of the Circle of Willis An artist's rendition of the normal anatomy of the Circle of Willis is shown. CCA: common carotid artery; ICA: internal carotid artery; ECA: external carotid artery; ACA: anterior cerebral artery; MCA: middle cerebral artery; ACOM: anterior communicating artery; PCA: posterior cerebral artery; PCOM: posterior communicating artery; BA: basilar artery; VA: vertebral artery

The normal origin of the ICA is from the common carotid artery. The ICA then traverses through the skull base and carotid sinus, arising into the dura. At the ICA terminus, the ICA leads into the middle cerebral artery (Figure 6) [5].

Agenesis of the ICA is a very rare congenital abnormality, with a reported incidence of 0.01% [3]. Agenesis or hypoplasia of the ICA occurs more frequently on the left side, with a ratio of three to one, between the left and right ICA involved [4]. The number of documented symptomatic cases is rare as the formation of collateral pathways provides sufficient cerebral perfusion.

Atypical congenital variants of the internal carotid arteries

There are six collateral circulation pathways described in the absence of the ICA [5].

Type A 

**Figure 7:**
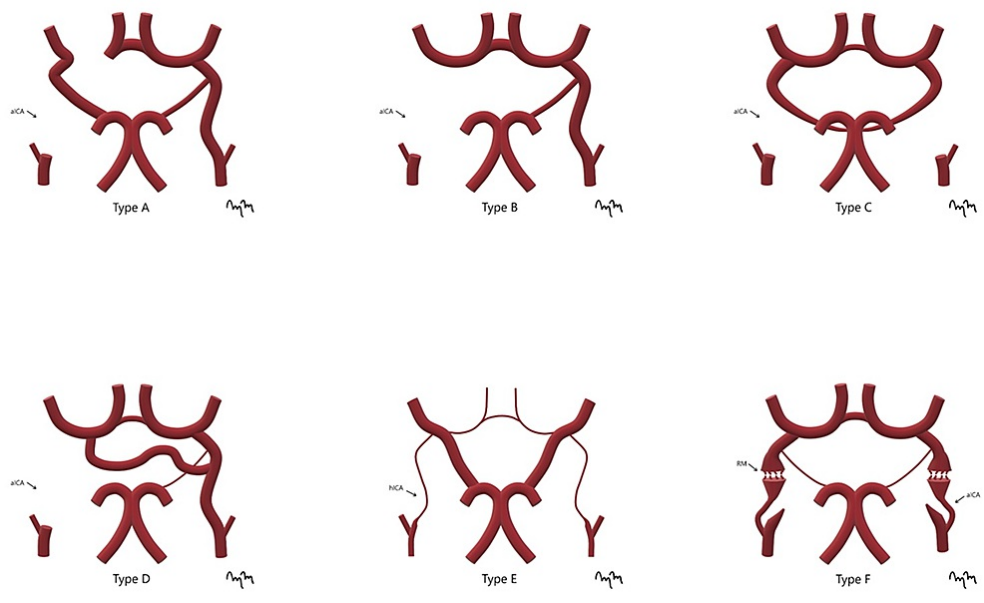
Six Types of Internal Carotid Artery Variants An artist's rendition of the six common internal carotid artery variants is shown. aICA: absent internal carotid artery; hICA: hypoplastic internal carotid artery; RM: rete mirabile

This consists of unilateral ICA agenesis with collateral circulation to the anterior circulation via the anterior communicating artery. There is collateral circulation to the ipsilateral middle cerebral artery via the posterior communicating artery (Figure 7).

Type B 

This consists of unilateral ICA agenesis with perfusion of both the ipsilateral anterior and middle cerebral arteries via a patent anterior communicating artery (Figure 7).

Type C

This consists of bilateral agenesis of the ICAs, with perfusion of the anterior circulation via the posterior vertebrobasilar circulation (Figure 7).

Type D

This consists of unilateral agenesis of the cervical portion of the ICA, with intercavernous anastomosis of the ipsilateral carotid siphon to the contralateral cavernous ICA (Figure 7).

Type E

This consists of bilateral hypoplasia of the ICAs that perfuse diminutive anterior cerebral arteries. Persistent hypertrophied posterior fetal circulation supplies the bilateral middle cerebral arteries (Figure 7).

Type F

This consists of bilateral transcranial anastomoses between the distal ICAs via internal maxillary branches from the external carotid arteries (Figure 7).

These six collateral pathways explained by Lie et al. have been further simplified into three groups [5]. The first and most common is collateral flow through the Circle of Willis, followed by collateral circulation through persistent embryonic circulation, and lastly the anastomosis of the ICA through the base of the skull by branches of the external carotid artery [6]. 

**Figure 8:**
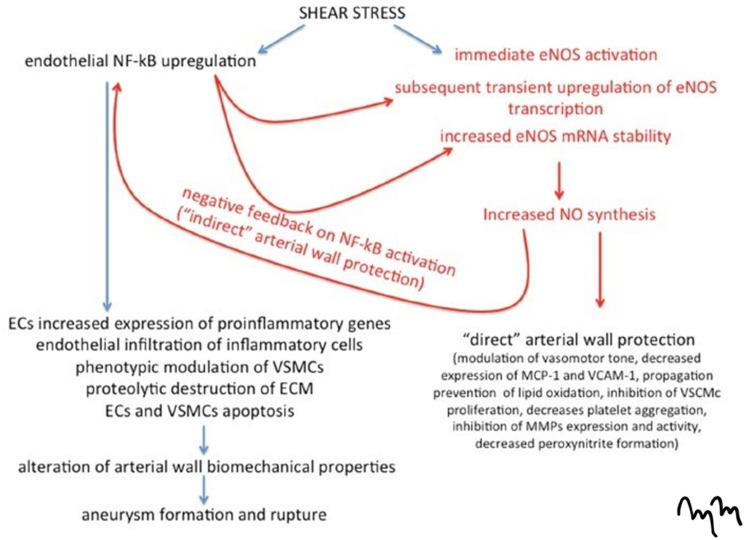
Pathophysiological Mechanism of Endothelial Wall Shear Stress The schematic demonstrates the activation of endothelial nitric-oxide synthase and endothelial nuclear factor kappa-B in high shear stress conditions on the arterial wall. The “direct” and “indirect” arterial wall protection is based on positive and negative feedback inhibition between stress‑mediated endothelial nuclear factor kappa B, cell upregulation, and nitric oxide synthesis. NF-kB: endothelial nuclear factor kappa-B; eNOS: endothelial nitric-oxide synthase; mRNA: messenger ribonucleic acid; NO: nitric oxide; ECs: endothelial cells; VSMCs: vascular smooth muscle cells; ECM: endothelial cell membranes; MCP-1: monocyte chemoattractant protein-1; VCAM-1: vascular cell adhesion molecule-1; VSCMc: vascular smooth cell membrane cytoplasm; MMPs: matrix metalloproteinases

The aberrant development of the ICA alters the vasculature of the anterior and posterior circulation, leading to altered flow dynamics. Patients who have congenital agenesis of the ICA of any kind are usually not symptomatic. However, the increased laminar flow through a singular ICA may cause shear stress injury to the arterial lumen, as a result of the increased flow burden. The shear stress caused by altered flow dynamics results in the upregulation of endothelial nuclear factor kappa B (NF-kB) and endothelial nitric oxide synthase (eNOS) through positive and negative feedback. The upregulation of NF-kB results in increased expression of pro-inflammatory markers from endothelial cells, proteolytic destruction of the extracellular matrix, and apoptosis of vascular smooth muscle cells and endothelial cells. This ultimately leads to aneurysm formation (Figure 8).

## Discussion

The first pharyngeal arch arteries classically involute, with a small percentage forming the maxillary arteries from remnants of the arch [1]. The second pharyngeal arch arteries also regress, with the dorsal aspects forming the stapedius artery [1]. The third pair of arteries form the common carotid arteries to supply the head [1]. Part of the aortic arch is formed by the left fourth pharyngeal arch arteries [1]. The right fourth pharyngeal arch artery becomes the proximal aspect of the right subclavian artery [1]. The fifth pair of pharyngeal arch arteries lack clinical importance, with 50% consisting of rudimentary vessels that eventually degenerate. Lastly, the sixth pharyngeal arch arteries develop into specific structures based on each location [1]. The proximal left side persists as the left pulmonary artery, and the distal part forms the ductus arteriosus. On the right, the proximal portion persists as the right pulmonary artery, and degenerates distally [1].

The derivatives of the third pharyngeal arch arteries are clinically important in light of the previously presented case. The proximal portions of the third pharyngeal arch arteries forms the common carotid arteries, and the distal portions join with the aortic knob to form the ICAs [2].

The ICA is divided into segments dependent on normal anatomy and development. The cervical segment (first) begins at the common carotid bifurcation and enters the skull via the carotid foramen. The ascending petrous segment (second) starts at the carotid foramen, travels to the anteromedial curvature of the petrous bone, and then ends at the caroticotympanic artery. The horizontal petrous segment (third) then extends to the foramen lacerum at the base of the skull. Next, the ascending cavernous segment (fourth) penetrates the cavernous sinus and ends at the origin of the meningohypophyseal trunk. The horizontal cavernous segment (fifth) continues within the cavernous sinus, from the meningohypophyseal trunk to the inferior-lateral trunk. The clinoidal segment (sixth) extends from the inferior-lateral trunk and is bordered distally by the origin of the ophthalmic artery. Finally, the last segment (seventh) of the ICA, lies between the ophthalmic artery and the anterior cerebral artery [7]. Deviation from these anatomical landmarks leads to variations and an aberrant ICA.

As described, the patient in our case had a type A ICA agenesis. The reasons she became symptomatic was from shear stress injury and increased turbulent flow resulting in upregulation of endothelial NF-kB and eNOS, ultimately leading to aneurysm formation.

The alterations in blood flow caused by congenital agenesis of the ICA are associated with intracranial vascular anomalies. The rate of intracranial aneurysms increases from 2-4% to 25-34% in patients with agenesis of an ICA [8]. This is thought to be the result of the increased hemodynamic forces placed on the vessels, due to the absence of the contralateral ICA. Another explanation may be secondary to an embryonic developmental error; however, this is less likely due to the prevalence of these aneurysms that are seen later in life, suggesting an acquired etiology [9].

Cerebral arteries lack an external elastic lamina, supporting perivascular tissue, and contain sparse medial elastin. This makes these vessels susceptible to local weakening and aneurysm formation [10]. Hemodynamic forces and wall shear stress are believed to be responsible for the development and growth of cerebral aneurysms. The high flow theory suggests that elevation in wall shear stress causes damage to the endothelium, initiating remodeling and the production of vasoactive substances [10]. The constant high wall shear stress leads to the overexpression of eNOS production. The local remodeling combined with increased flow volume and pressure leads to the local dilation of the arterial wall, and subsequent aneurysm formation, propagation, and rupture. Other key considerations include atherosclerotic and thromboembolic diseases, known to result from hemodynamic disturbances as well.

Symptoms caused by agenesis of the ICA with subsequent contralateral aneurysm formation are dependent on the severity, growth, location, and/or aneurysm rupture. Symptoms can include headache, blurry vision, hemiplegia, transient ischemic episodes, and intracranial hemorrhage.

## Conclusions

Agenesis or dysgenesis of the ICA is rare, and may never clinically manifest. However, under the appropriate conditions, complex physiologic mechanisms may result in the general increase in shear stress upon the contralateral ICA, leading to aneurysm formation and impending rupture. The patient from the preceding case had agenesis of the left ICA (type A), which lead to the aneurysmal formation and rupture of the contralateral ICA.
